# Prevalence and antimicrobial susceptibility patterns of bacteria colonizing the external ocular surfaces of patients undergoing ocular surgeries at Bugando Medical Center in Mwanza, Tanzania

**DOI:** 10.1186/s13104-024-06851-3

**Published:** 2024-07-11

**Authors:** Johannes Rukyaa, Lillian Temba, Princekened Kachira, Christopher Mwanansao, Jeremiah Seni

**Affiliations:** 1 Department of Microbiology and Immunology, Weill Bugando School of Medicine, Catholic University of Health and Allied Sciences, P. O. Box 1464, Mwanza, Tanzania; 2 Department of Surgery, Weill Bugando School of Medicine, Catholic University of Health and Allied Sciences, P. O. Box 1464, Mwanza, Tanzania; 3 Department of Ophthalmology, Bugando Medical Centre, P.O.Box 1370, Mwanza, Tanzania

**Keywords:** Ocular surgeries, Bacterial colonization, Bugando medical centre, Tanzania

## Abstract

**Objective:**

Understanding microbiota colonizing ocular surfaces is key to expedite antibiotic prophylactic options for ocular surgeries, and therefore, prevent subsequent surgical site infections (SSIs). To fill this critical gap, we aimed at determining the prevalence and antibiotic susceptibility patterns of bacteria colonizing the external ocular surfaces of 224 patients undergoing ocular surgeries at Bugando Medical Centre (BMC) in Mwanza, Tanzania between May and August 2023.

**Results:**

The study participants had a median age of 62.5 (interquartile range: 39.5–75.0) years. A total of 78.1% (175/224) ocular swabs were culture positive yielding 196 bacterial isolates. *Staphylococcus epidermidis* [43.4% (*n* = 85)], *Staphylococcus aureus* [21.9% (*n* = 43)] and *Pseudomonas aeruginosa* [14.3% (*n* = 28)] were the most common bacteria. There were low proportions of resistance among predominant Gram-positive and Gram-negative bacteria to gentamicin (≤ 25.0%), and similarly, low resistance among Gram negative bacteria was observed against 3rd generation cephalosporins (≤ 25.0%) and piperacillin-tazobactam (0.0%). Variable resistance profiles were notable to the most commonly used antibiotics (ciprofloxacin and tetracycline: 0.0–66.7%). Our findings underscore an urgent need to revisit antibiotic prophylactic guidelines for ocular surgeries in this tertiary hospital, and calls for prospective evaluation of incident SSIs post-ocular surgeries to guide specific management.

## Introduction

Surgical site infections (SSI) are defined as infections occurring within 30 days after a surgical procedure affecting the superficial or deep tissue at the operation site [1]. A study conducted in the United States of America assessing the role of topical antibiotics prophylaxis in oculofacial plastic surgery reported ocular surgical site infections (OSSI) to be more common in the placebo group 2.7% than the antibiotic group 0.0% [2]. Likewise, a study conducted in Brazil reported higher rates of postoperative endophthalmitis in the control group (0.097%) than the group receiving antibiotic prophylaxis (0.018%) [3].

The OSSI predispose patients to increased risk of prolonged post operative hospital stay, lifelong disability, and increased cost of patients management [1, 4]. Infectious postoperative endophthalmitis (IPOE) is the most dreaded complication for ocular surgical procedures [5]. The severity and clinical outcome of OSSI (including IPOE) depends on the pathogens’ virulence and inoculum, patient’s immunological status, time of diagnosis and treatment, and other external/environmental factors [6, 7].

In most cases of SSI, the causative pathogens are the normal microbiota inhabiting the respective anatomical surgical sites [8], and knowing the types and patterns of these bacteria is pertinent in expediting prophylactic antibiotics, and therefore, decrease the risk of acquiring SSI [9]. In this regard, the most targeted bacterial species for provision of antibiotic prophylaxis are Gram positive bacteria such as *Staphylococcus aureus, Staphylococcus epidermidis* and other Coagulase-negative Staphylococci (CoNS). Various Streptococci and Enterococci species are also of significant importance [7, 10, 11]. The continuous shift of ocular microbial flora due to factors such as age, occupation, place of residence, co-existing morbidities, and previous history of antibiotic use and hospital admission (to mention a few), may challenge the existing pre-operative prophylactic guidelines and accelerate the emergence of antimicrobial resistance (AMR) [12, 13].

Therefore; this study was designed to evaluate etiologies and antibiotic susceptibility patterns of bacteria residing on the external ocular surface of pre-operative surgical patients so as to come up with an up-to-date information that can be used to formulate prophylactic guidelines based on local evidence.

## Methods

### Study design, duration and setting

This was a hospital based cross sectional analytical study conducted from May 2023 to August 2023 in the Ophthalmology Department at Bugando Medical center (BMC). The BMC is a 1000-bed capacity zonal tertiary consultant and teaching hospital serving as a referral center for over 20 million people in the north-western part of Tanzanian [14, 15]. The Ophthalmology Department has a bed capacity of 50, it attends approximately 400 patients monthly in the outpatient clinics, and performs approximately 80 ocular surgeries per month.

### Study population, selection criteria and sampling

This study included all patients presenting at the Ophthalmology Department at BMC for ocular surgeries, and excluded all patients who presented with signs and symptoms of active ocular infections. Using a Kish-Leslie formula for cross-sectional studies, and a proportion of conjunctival bacterial colonization of 26.6% from a previous study in Taiwan among patients undergoing cataract surgery, a minimum sample size of 300 patients were anticipated to be enrolled [16, 17]. However, during the study period a total of 240 patients were enrolled. A total of 16 patients were excluded either due to missing data or had an active eye infection, bringing the number of study participants to 224 patients.

### Data collection and laboratory procedures

A pretested-structured questionnaire was developed and then, fed into the Epi-collect- 5 software^®^ (Centre for Genomic Pathogen Surveillance, UK) and subsequently used to collect socio-demographic and clinical information from patients. Each patient was informed about the study procedures, risks, benefits and rights to participate or withdrawal from the study, and then, those who voluntarily agreed to participate were enrolled and their respective data and samples collected. Ocular swabs were aseptically collected by the principal investigator (or a trained research assistant) from each patient in the morning on the day of the surgical procedure before the application of topical anesthetic, mydriatics, antibiotic or povidone-iodine. The patient was asked to look up, and then the eyelid margin was swabbed with a sterile cotton swab moistened with sterile saline, employing a continuous stroke from the nasal to temporal side and then a second stroke from temporal to nasal side without touching the eyelashes, and then, placed in the swab’s transport media (Bio lab, HUNGARY^®^). Samples were transported to the Clinical Microbiology Laboratory of the Catholic University of Health and Allied Sciences in the cool box. In the laboratory, the swabs from patients were inoculated into Brain-heart infusion (BHI) broth (OXOID, Hampshire, United Kingdom) for enrichment and then processed in the laboratory after 18–24 h incubation at 35-37^o^C.

### Culture, bacterial identification and drug susceptibility testing

A portion of homogenized BHI was obtained using a sterile wire loop and inoculated onto blood agar (BA) and MacConkey agar (MCA) (OXOID, Hampshire, United Kingdom) incubated for 18–24 h at 35–37°c. Identification of the organisms was done based on the growth characteristics on BA, MCA, secondary gram stain, and biochemical characteristics [18].

A single colony of bacteria from a fresh pure culture plate was emulsified into sterile normal saline to achieve a concentration equivalent to 0.5 McFarland turbidity solution, a sterile cotton swab was then used to obtain bacteria from the suspension, the swab was then squeezed against the wall of the tube to remove excess fluid before being seeded uniformly onto Muller-Hinton agar (OXOID, Hampshire, United Kingdom) plate. Antibiotics of the right potency was placed on the agar to test for antibiotic susceptibility patterns using Kirby Bauer’s disc diffusion method as guided by the Clinical Laboratory Standard Institute (CLSI) 30th edition M100 document, 2020 [19].

Antibiotics discs for Gram-positive bacteria included: ampicillin(10 µg), cefoxitin(30 µg), trimethoprim-sulfamethoxazole (1.25/23.75 µg), ciprofloxacin (5 µg), erythromycin (15 µg), clindamycin (2 µg), vancomycin (30 µg), linezolid (30 µg), gentamicin (10 µg), clindamycin (2 µg), tetracycline (30 µg), whereas the antibiotics discs for Gram-negative bacteria were: ceftriaxone (30 µg), ceftazidime(30 µg), cefepime (30 µg), ciprofloxacin (5 µg), piperacillin-tazobactam (100/10µg), amoxicillin/clavulanate (20/10µg), trimethoprim-sulfamethoxazole (1.25/23.75 µg), meropenem (10 µg), gentamicin (10 µg) and tetracycline (30 µg).

All *S. aureus* and *S. epidermidis* with a zone of inhibition on cefoxitin (30 µg) disc of ≤ 21 mm were regarded as methicillin resistant *S. aureus* (MRSA) and methicillin resistant coagulase negative *Staphylococcus* (MRCoNS), respectively. Inducible clindamycin resistance was tested by observing the blunting of the zone of inhibition around the clindamycin disc placed adjacent to the erythromycin disc. For gram negative bacteria; extended spectrum beta lactamase (ESBL) phenotype was confirmed using combined disc method using cefotaxime 30 µg disk and cefotaxime 30 µg combined with clavulanic acid 10 µg disk [20].

### Quality control

Aseptic techniques were strictly observed during sample collection, transportation and processing. Control strains of *Staphylococcus aureus* (ATCC 25,923) and *Escherichia coli* ( ATCC 25,922) were used for quality control of the performance of the culture media and the antibiotic discs for Gram-positive and Gram-negative bacteria, respectively [20].

### Statistical analysis and ethical consideration

Excel data sheet was extracted from Epi-collect- 5 software^®^ and then, laboratory data were also added into the Microsoft Excel. Data was transferred to STATA version15 (College Station, Texas, USA) for analysis. Continuous data was summarized using medium and inter-quartile range (IQR). Categorical data was summarized using proportion (percent). Pearson chi squared test (or 1-sided Fisher’s exact where applicable) and a two-sample test of proportions was used to assess the distribution of categorical variables against ocular colonization culture positivity. A *p*-value cut-off of less than 0.05 was considered statistically significant. This study obtained an ethical clearance from the joint CUHAS/BMC Research Ethics Review Committee (CREC 2900/2023). Permission to conduct the study was obtained from the BMC Hospital Director General, whereas voluntary informed consent from participants was obtained before proceeding to data and sample collection. Confidentiality of the patients was observed throughout the study. Final results from this study were communicated to the Department of Ophthalmology to guide future revision of antimicrobial prophylaxis guidelines.

## Results

### Socio-demographic information of the study participants

This study enrolled a total of 224 patients, majority of the study participants were male 54.5% (*n* = 122). The median age of the study participants was 62.5 [IQR 39.5–75.0] years with the majority of the study participants being self-employed 50.0% (*n* = 122) and had attended primary education level 47.3% (*n* = 106), Table 1.

**Table 1:** Social-demographic characteristics of study participants

Variables	Category	Frequency (%)
Gender	Male	122(54.5)
	Female	102 (45.5)
Marital status	Divorced	9 (4.0)
	Married	120 ( 53.6)
	Not married	47 (21.0)
	Widow	48 (21.4)
Education level	Primary	106 (47.3)
	Secondary	43 (19.2)
	Tertiary	24 (10.7)
	Never attended school	51 (22.8)
Occupation	Employed	17 (7.6)
	Self employed	112 (50.0)
	Retired	65 (29.0)
	Student	30 (13.4)
Ward	Female ward	97 (43.3)
	Male ward	107 (47.8)
	Pediatric ward	20 (8.9)

Majority of the study participants underwent cataract removal 57.6% (*n* = 129). Comorbidities were observed in 34.8% (*n* = 78) of study participants, and high blood pressure predominated, 19.2% (*n* = 43) as shown in Table 2.

**Table 2:** Clinical characteristics of study participants

Variables	Category	Frequency (%)
Type of ocular procedure	Cataract removal	129 (57.6)
	Glaucoma	2 (0.9)
	Intraocular surgeries	93 (41.5)
Antibiotic consumption	No	221 (98.7)
	Yes	3 (1.3)
Type of antibiotic used	Ceftriaxone	1 (0.5)
	Ampicillin-cloxacillin	2 (0.8)
	N/A	221 (98.7)
Mode of antibiotic acquisition	Self-prescription	2 (0.8)
	Doctor’s prescription	1 (0.5)
	N/A	221 (98.7)
Duration of antibiotic use	2(days)	2 (0.8)
	3(days)	1 (0.5)
	N/A	221 (98.7)
Comorbidities	No	146 (65.2)
	Yes	78 (34.8)
Type of comorbidities	High blood pressure	43 (19.2)
	Diabetes mellitus	10 (4.5)
	Peptic ulcers	9 (4.0)
	Others**	16 (7.1)
	None	146 (65.2)

** Unspecified ear problem (3), Cardiomegaly (3), Joint pain (3), Unspecified febrile illness (2) Prostate cancer (1), Leg pain (1), Kidney problem (1), Hernia (1), and *Tuberculosis (1)*

### Prevalence of bacteria colonizing external ocular surfaces

Out of 224 non repetitive ocular swabs collected, 175 (78.1%) were culture positive for bacteria colonizing external ocular surfaces. A total of 21 (9.4%) of the study participants had positive culture for more than one bacterial species, resulting into a total of 196 bacteria isolates. Culture positivity was significantly higher among patients with comorbidities compared to those who without comorbidities (85.9% versus 74.0%, *p* = 0.040) Table 3.

**Table 3:** Study variables vs. culture positivity among study participants

Variables	Category	Culture results		Pearson chi^2^ test	*p*-value
		**Positive**, **n (%)**	**Negative**, **n (%)**		
Gender	Male	100(82.0)	22 (18.0)	2.314	0.128
	Female	75 (73.5)	27 (26.5)		
Age category (years)	≤ 50	48 (72.7)	18 (27.3)	1.5952	0.207
	> 50	127 (80.4)	31 (19.6)		
Education level	Primary	80 (75.5)	26 (24.5)	2.595	0.458
	Secondary	35 (81.4)	8 (18.6)		
	Tertiary	17 (70.8)	7 (29.2)		
	Never attended school	43 (84.3)	8 (15.7)		
Occupation	Employed	9 (52.9)	8 (47.1)	7.397	0.060
	Self employed	89 (79.5)	23 (20.5)		
	Retired	54 (83.1)	11 (16.9)		
	Student	23 (76.7)	7 (23.3)		
Ocular procedure	Cataract removal	32 (24.2)	97 (75.8)	-	0.173*
	Glaucoma	1 (50.0)	1 (50.0)		
	Intraocular surgeries	77 (82.8)	16 (17.2)		
Comorbidities	No	108 (74.0)	38 (26.0)	4.230	0.040
	Yes	67(85.9)	11 (14.1)		

*p-value based on one-sided Fisher’s exact

### Distribution of bacterial isolates colonizing external ocular surfaces and their antimicrobial susceptibility patterns

A total of 196 bacteria were isolated from non-repetitive ocular swabs in this study, *S. epidermidis, S. aureus*, and *P. aeruginosa* were the commonest isolates: 43.4% (*n* = 85), 21.9% (*n* = 43) and 14.3% (*n* = 28), respectively. Low level resistance among *S. epidermidis* and *S. aureus* were observed against clindamycin (28.2% and 27.9%, respectively) and gentamicin (14.1% and 16.3%, respectively). For the predominant Gram-negative bacteria, *P. aeruginosa* and *Klebsiella* spp., showed low level resistance was observed against ceftazidime at 21.4% and 12.5%, respectively. All Gram-negative bacteria were sensitive to meropenem except one *Klebsiella pneumoniae* isolate. Variable resistance profiles were notable to the most commonly used antibiotics like ciprofloxacin and tetracycline, ranging from 0.0 to 66.7% (Table 4).

This study observed high proportion of MRCoNS and MRSA at 52.9% and 46.5%, respectively although the difference was not statistically significant (*p* = 0.494). There was low ESBL production among Gram negative Enterobacterales (15.4%, 4/26), Table 3.

### Distribution of major AMR phenotype by age, gender and pre-operative diagnosis

The prevalence Methicillin-resistant Staphylococci ocular colonization among study participants was 29.5% (66/224), whereas the prevalence of cephalosporin resistant Gram-negative bacterial ocular colonization was 5.4% (12/224). There was no statistical difference in distribution of Methicillin-resistant Staphylococci ocular colonization among participants less than 50 years of age compared to those older than 50 years (24.2%, 16/66 versus 31.7%, 50/158, respectively, *p*-value = 0.268). Similarly, the distribution of Methicillin-resistant Staphylococci ocular colonization between male and female were 32.0% (39/122) and 26.5% (27/102), respectively; *p*-value = 0.369. On the other hand, the distribution of cephalosporin resistant Gram-negative bacterial ocular colonization among participants less than 50 years of age and in those older than 50 years were 4.6% (3/66) and 5.7% (9/158), respectively, 1-sided Fisher’s exact *p*-value = 0.507). The distribution of cephalosporin resistant Gram-negative bacterial ocular colonization between male and female were 6.6% (8/122) and 3.9% (4/102), respectively; 1-sided Fisher’s exact *p*-value = 0.286.

Of note, Methicillin-resistant Staphylococci ocular colonization was significantly more among participants undergoing cataract removal surgeries compared to those undergoing intraocular surgeries (26.4%, 34/129 versus 14.0%, 13/93, respectively, *p*-value = 0.026). There were low proportions of cephalosporin resistant Gram-negative bacterial ocular colonization in patients undergoing cataract removal and intraocular surgeries (3.1%, 4/129 and 8.6%, 8/93, respectively; *p*-value = 0.074), but the difference was not statistically significant.

**Table 4:** Antibiotic susceptibility patterns of bacteria isolated from patients

Bacteria, *n* (%)	Antibiotics susceptibility patterns													
		CIP(%)	TET(%)	E (%)	CD (%)	COT(%)	FOX (%)	GEN (%)	LZ (%)	FEP (%)	CRO (%)	CAZ (%)	AMC(%)	TZP (%)
*S.epidermidis*, 85 (43.4)	***S***	47(55.2)	32(37.6)	25(29.4)	61(71.8)	11(12.9)	40(47.1)	73(85.9)	84(98.8)	NA	NA	NA	NA	NA
	***R***	38(44.8)	53(62.4)	60(70.6)	24(28.2)	74(87.1)	45(52.9)	12(14.1)	1(0.2)	NA	NA	NA	NA	NA
*S. aureus*, 43 (21.9)	***S***	29(67.4)	16(37.2)	14(32.6)	31(72.16)	11(25.6)	23(53.5)	36(83.7)	42(97.7)	NA	NA	NA	NA	NA
	***R***	14(32.6)	27(62.8)	29(67.4)	12(27.9)	32(74.4)	20(46.5)	7(16.3)	1(2.3)	NA	NA	NA	NA	NA
*P. aeruginosa, 28*(14.3)	***S***	28(100)	NA	NA	NA	NA	NA	26(92.9)	NA	27(96.4)	NA	22(78.6)	NA	27(96.4)
	***R***	0 (0.0)	NA	NA	NA	NA	NA	2(7.1)	NA	1(3.6)	NA	6(21.4)	NA	1 (3.6)
*Klebsiella* spp, 24(12.2)	***S***	21(87.5)	24(100)	NA	NA	22(91.7)	NA	22(91.7)	NA	21(87.5)	21(87.5)	21(87.5)	7(29.2)	24(100.0)
	***R***	3(12.5)	0 (0.0)	NA	NA	2(8.3)	NA	2(8.3)	NA	3(12.5)	3(12.5)	3(12.5)	17(70.8)	0 (0.0)
S. *viridans*, 9 (4.6)	***S***	3(33.3)	5(55.5)	5(55.5)	8(88.9)	1(11.1)	NA	4(45.5)	9(100)	NA	NA	NA	NA	NA
	***R***	6(66.7)	4(45.5)	4(45.5)	1(11.1)	8(88.9)	NA	5(55.5)	0 (0.0)	NA	NA	NA	NA	NA
Other GNB*, 3 (1.5)	***S***	2(66.7)	2(66.7)	NA	NA	2(66.7)	NA	2(66.7)	NA	2(66.7)	2(66.7)	2(66.7)	0 (0.0)	3(100)
	**R**	1(33.3)	1(33.3)	NA	NA	1(33.3)	NA	1(33.3)	NA	1(33.3)	1(33.3)	1(33.3)	3(100)	0(0.0)
**Total**, **196 (100.0)**														

*Escherichia coli (2), *Acinetobacter* spp (1)

**Note**: CIP: Ciprofloxacin, TET: Tetracycline, E: Erythromycin, CD: Clindamycin, FOX: Cefoxitin, GEN: Gentamicin,

COT: Trimethoprim-sulfamethoxazole, LZ: Linezolid, FEP: Cefepime, CRO: Ceftriaxone, CAZ: Ceftazidime,

AMC: Amoxicillin/clavulanate, TZP: Piperacillin-tazobactam, NA: Not Applicable, R: Resistant; S: Sensitive

## Discussion

This study enrolled a total of 224 patients who were attending the Ophthalmology Department at BMC with the majority of participants being male, similar to other studies [21, 22]. This study observed high proportion for ocular surface colonization by bacteria (78.1%), than 59.5% and 32.5% reported from a study conducted at a tertiary hospital in Uganda in 2013 and another study conducted in Mexico in 2021, respectively [23, 24], however; our study findings are lower than 85% reported from USA in 2015 [25]. These high proportions reported in both studies are indicative of the high proportion colonization in the external ocular surfaces of patients undergoing ocular surgeries due to the fact that the eye lid margins mark the border between the external and internal ocular surface, therefore, being in close proximity with the skin which harbors high loads of skin microbiota [26, 27].

This study reported predominance of Gram-positive skin microbiota (*S. epidermidis* and *S. aureus*) colonizing external ocular surfaces, which is not surprising and is in agreement with studies from Uganda and the USA [23, 24]. It is well known that these skin microbiota are preferentially important in preventing pathogenic bacteria from causing OSSIs, however, during ocular surgeries, they can be introduced into internal ocular structures and potentially cause OSSIs if appropriate antibiotic prophylaxis are not administered [28, 29]. Furthermore; we observed the unusual isolation of Gram negative pathogenic bacteria like *P. aeruginosa* and *Klebsiella* spp. from the external ocular surfaces, similar to findings from a study in Mexico [24]. These findings may be attributed to contamination from various inanimate/environmental surfaces and patients anatomical sites like the perianal areas [30, 31]. Therefore, this calls for strengthening of hygienic and sanitation practices among patients, and blocking this transmission cycle [32]. The findings are further reiterated by similar occurrence of these pathogens in other hospital environmental premises at BMC which are linked to neonatal sepsis and neonatal deaths, and therefore, a need to strengthen hospital-wide infection prevention and control measures through environmental cleaning and decontamination [33, 34]. Ou findings highlight a need to conduct prospective study in patients undergoing ocular surgeries to evaluate potential involvement of these pathogens in the OSSIs, and expedite specific antimicrobial therapies.

High proportion of resistance of *S. epidermidis* and *S. aureus* was observed towards commonly used antibiotics like trimethoprim/sulfamethoxazole, erythromycin and tetracycline, unlike less commonly used agents like gentamicin. Similarly, *Klebsiella* spp. displayed high proportion of resistance towards amoxicillin/clavulanic acid, compared to less commonly used agents like gentamicin, 3rd generation cephalosporin and piperacilllin-tazobactam. These findings provided a wake-up calls as antibiotics like erythromycin and tetracycline are commonly used at BMC Ophthalmology Department as prophylaxis for ocular surgical patients, and in other lower- and middle-income countries (LMIC’s) settings [35, 36]. Therefore, the findings are pertinent to guide revisiting of antibiotic prophylactic guidelines for ocular surgeries at BMC in Mwanza, Tanzania.

This study observed high proportions of MRCoNS and MRSA in *S. epidemidis* and *S. aureus* isolates, respectively among pre-operative patients posing a risk for subsequent OSSIs attributed to these notorious and difficult to manage strains. The risky was significantly more among participants undergoing cataract removal surgeries than to those undergoing intraocular surgeries, rendering cloxacillin/flucloxacillin therapies ineffective in the former group of patients. This may further lead to prolonged hospitalization, lifelong disability and an increased cost for patient care [37]. The findings highlight an emerging antimicrobial selective pressure not only to pathogens but also to normal microbiota, and hence, a need to escalate our responsive measures towards AMR surveillance and antimicrobial stewardship programs at BMC [38]. On the other hand, there was low resistance to the 3rd generation cephalosporins marked by ESBL production in Gram negative Enterobacterales, emphasizing a need to foster rational use of these agents and maintain their efficacy for prophylaxis and therapeutic roles. We observed that having comorbidity was a risk factor for ocular colonization. This is likely to be associated with recurrent exposure to hospital environment (outpatient visits and hospital admissions) and antibiotic exposure, which altogether are known to be driving factors for AMR. Therefore, priority screening for patients with comorbidities is emphasized to as to guide specific choice of antibiotic prophylactic agents.

## Conclusions

Approximately eight out of every ten patients undergoing ocular surgical procedures at BMC are colonized by normal microbiota and pathogenic bacteria in their external ocular surfaces. These bacteria display high proportions of resistance towards erythromycin, tetracycline, and ciprofloxacin which are commonly used as prophylaxis for ocular surgeries in this setting. Gentamicin, 3rd generation cephalosporins and piperacillin-tazobactam showed low resistance (≤ 25.0%), and are potential antimicrobial prophylactic options in this hospital upon revisiting hospital guidelines. Prospective studies at BMC should focus on evaluating relationship between ocular colonization and incident OSSIs, as well as transmission dynamics of these pathogens between patients and hospital environmental premises using genomic approaches are reiterated.

### Limitations

This study employed conventional culture techniques which might have missed the isolation of difficult to culture bacteria pathogens, and therefore, may have under reported the actual burden for bacterial ocular colonization. This study did not asses the relationship between ocular colonization and incident OSSIs, which may be a pivotal area of interest for future studies at BMC.

## Data Availability

Dataset analyzed to generate study findings is available to the corresponding author on request.

## Abbreviations

AMR: Antimicrobial resistance
ATCC: American type culture collection
BA: Blood agar
BHI: Brain-heart infusion
BMC: Bugando Medical center
CoNS: Coagulase-negative staphylococci
CUHAS: Catholic University of Health and Allied Sciences
ESBL: Extended Spectrum Beta Lactamase
IPOE: Infectious postoperative endophthalmitis
MCA: MacConkey agar
OSSI: Ocular surgical site infections
SSI: Surgical site infections

## References

[1] Owens C, Stoessel K (2008). Surgical site infections: epidemiology, microbiology and prevention. J Hosp Infect.

[2] Ashraf DC, Idowu OO, Wang Q, YeEun T, Copperman TS, Tanaboonyawat S, Arnold BF, Oldenburg CE, Vagefi MR, Kersten RC (2020). The role of topical antibiotic prophylaxis in oculofacial plastic surgery: a randomized controlled study. Ophthalmology.

[3] de Sousa Casavechia LN, Meireles AC, Schapira E, Fernandes RAB, Fernandes AG (2023). The impact of antibiotic prophylaxis with intracameral cefuroxime on postoperative infectious endophthalmitis rates in a high-volume cataract surgery center. Sci Rep.

[4] Reichman DE, Greenberg JA (2009). Reducing surgical site infections: a review. Reviews Obstet Gynecol.

[5] Behndig A, Cochener B, Güell JL, Kodjikian L, Mencucci R, Nuijts RM, Pleyer U, Rosen P, Szaflik JP, Tassignon M-J (2013). Endophthalmitis prophylaxis in cataract surgery: overview of current practice patterns in 9 European countries. J Cataract Refractive Surg.

[6] Lee PJ, Seal DV, Payman G. Endophthalmitis: diagnosis and management. CRC Press LLC; 2004.

[7] Barry P, Behrens-Baumann W, Pleyer U, Seal D (2007). ESCRS guidelines on prevention, investigation and management of post-operative endophthalmitis. Version.

[8] Khairy GA, Kambal AM, Al-Dohayan AA, Al-Shehri MY, Zubaidi AM, Al-Naami MY, AlSaif FA, Al-Obaid OA, Al-Saif AA, El-Farouk OY (2011). Surgical site infection in a teaching hospital: a prospective study. J Taibah Univ Med Sci.

[9] Bowater RJ, Stirling SA, Lilford RJ (2009). Is antibiotic prophylaxis in surgery a generally effective intervention? Testing a generic hypothesis over a set of meta-analyses. Ann Surg.

[10] Group EVS (1995). Results of the endophthalmitis vitrectomy study. Arch Ophthalmol.

[11] Fisch A, Prazuck T, Gerbaud L, Coscas G, Lafaix C, Salvanet A, Forestier F. Endophthalmitis FCSGo: Epidemiology of infective eridophthalmitis in France. The Lancet. 1991;338(8779):1373–1376. 1682746

[12] Storey P, Dollin M, Rayess N, Pitcher J, Reddy S, Vander J, Hsu J, Garg S, Team P-IES (2016). The effect of prophylactic topical antibiotics on bacterial resistance patterns in endophthalmitis following intravitreal injection. Graefe’s Archive Clin Experimental Ophthalmol.

[13] Malik B, Bhattacharyya S (2019). Antibiotic drug-resistance as a complex system driven by socio-economic growth and antibiotic misuse. Sci Rep.

[14] Clinical Services Updates. [https://www.bugandomedicalcentre.go.tz/index.php?bmc=61].

[15] United Republic of Tanzania. National Populationa and Housing Census. 2022.

[16] Lin YH, Kang YC, Hou CH, Huang YC, Chen CJ, Shu JC, Hsieh PH, Hsiao CH (2017). Antibiotic susceptibility profiles of ocular and nasal flora in patients undergoing cataract surgery in Taiwan: an observational and cross-sectional study. BMJ Open.

[17] Kish L. Sampling organizations and groups of unequal sizes. Am Sociol Rev 1965:564–72. 14325826

[18] Koneman EW, Allen SD, Janda W, Schreckenberger P, Winn W. Diagnostic microbiology. The nonfermentative gram-negative bacilli Philedelphia: Lippincott-Raven Publishers. 1997:253–320.

[19] Jorgensen JH, Turnidge JD. Susceptibility test methods: dilution and disk diffusion methods. Man Clin Microbiol 2015:1253–73.

[20] CLSI: Clinical Laboratory Standard Institute. CLSI M100-ED29: 2021 Performance Standards for Antimicrobial Susceptibility Testing, 30th Edition. 2020;40. In.; 2020.

[21] La Gonzalez-De A, Franco-Cendejas R, Gonzalez-Veliz A, Zarza-Garcia V, ML GH, Barrientos-Gutierrez T (2022). Ocular surface characteristics and colonization in a burn Center: a prospective cohort study. J Burn Care Research: Official Publication Am Burn Association.

[22] Zhao H, Chen Y, Zheng Y, Xu J, Zhang C, Fu M, Xiong K (2023). Conjunctival sac microbiome in anophthalmic patients: Flora diversity and the impact of ocular prosthesis materials. Front Cell Infect Microbiol.

[23] Mshangila B, Paddy M, Kajumbula H, Ateenyi-Agaba C, Kahwa B, Seni J (2013). External ocular surface bacterial isolates and their antimicrobial susceptibility patterns among pre-operative cataract patients at Mulago National Hospital in Kampala, Uganda. BMC Ophthalmol.

[24] De La Parra-Colin P, Gonzalez-De La Torre A, Franco-Cendejas R, Gonzalez-Veliz A, Zarza-Garcia V (2022). Vázquez Mellado Martínez IP, García Hernández MdL, Barrientos-Gutierrez T: ocular surface characteristics and colonization in a burn center: a prospective cohort study. J Burn Care Res.

[25] Jassim SH, Sivaraman KR, Jimenez JC, Jaboori AH, Federle MJ, de la Cruz J, Cortina MS (2015). Bacteria colonizing the ocular surface in eyes with boston type 1 keratoprosthesis: analysis of biofilm-forming capability and Vancomycin tolerance. Investig Ophthalmol Vis Sci.

[26] Brown MM, Horswill AR (2020). Staphylococcus epidermidis—skin friend or foe?. PLoS Pathog.

[27] Parlet CP, Brown MM, Horswill AR (2019). Commensal staphylococci influence Staphylococcus aureus skin colonization and disease. Trends Microbiol.

[28] Severn MM, Horswill AR (2023). Staphylococcus epidermidis and its dual lifestyle in skin health and infection. Nat Rev Microbiol.

[29] Krishna S, Miller LS (2012). Host–pathogen interactions between the skin and Staphylococcus aureus. Curr Opin Microbiol.

[30] Erb S, D’Mello-Guyett L, Malebo HM, Njee RM, Matwewe F, Ensink J, Hinic V, Widmer A, Frei R (2018). High prevalence of ESBL-Producing E. Coli in private and shared latrines in an informal urban settlement in Dar Es Salaam, Tanzania. Antimicrob Resist Infect Control.

[31] Silago V, Kovacs D, Msanga DR, Seni J, Matthews L, Oravcová K, Zadoks RN, Lupindu AM, Hoza AS, Mshana SE (2020). Bacteremia in critical care units at Bugando Medical Centre, Mwanza, Tanzania: the role of colonization and contaminated cots and mothers’ hands in cross-transmission of multidrug resistant gram-negative bacteria. Antimicrob Resist Infect Control.

[32] Bloomfield SF, Exner M, Signorelli C, Nath K, Scott E. The chain of infection transmission in the home and everyday life settings, and the role of hygiene in reducing the risk of infection. In: International scientific forum on home hygiene: 2012; 2012.

[33] Marando R, Seni J, Mirambo MM, Falgenhauer L, Moremi N, Mushi MF, Kayange N, Manyama F, Imirzalioglu C, Chakraborty T (2018). Predictors of the extended-spectrum-beta lactamases producing Enterobacteriaceae neonatal sepsis at a tertiary hospital, Tanzania. Int J Med Microbiol.

[34] Kovacs D, Silago V, Msanga DR, Mshana SE, Seni J, Oravcova K, Matthews L (2022). The hospital environment versus carriage: transmission pathways for third-generation cephalosporin-resistant bacteria in blood in neonates in a low-resource country healthcare setting. Sci Rep.

[35] Kariyawasam RM, Julien DA, Jelinski DC, Larose SL, Rennert-May E, Conly JM, Dingle TC, Chen JZ, Tyrrell GJ, Ronksley PE (2022). Antimicrobial resistance (AMR) in COVID-19 patients: a systematic review and meta-analysis (November 2019–June 2021). Antimicrob Resist Infect Control.

[36] Ndaki PM, Mushi MF, Mwanga JR, Konje ET, Ntinginya NE, Mmbaga BT, Keenan K, Sabiiti W, Kesby M, Benitez-Paez F (2021). Dispensing antibiotics without prescription at community pharmacies and accredited drug dispensing outlets in Tanzania: a cross-sectional study. Antibiotics.

[37] Serra-Burriel M, Keys M, Campillo-Artero C, Agodi A, Barchitta M, Gikas A, Palos C, López-Casasnovas G (2020). Impact of multi-drug resistant bacteria on economic and clinical outcomes of healthcare-associated infections in adults: systematic review and meta-analysis. PLoS ONE.

[38] Beyer P, Paulin S (2020). Priority pathogens and the antibiotic pipeline: an update. Bull World Health Organ.

